# What Managers Find Important for Implementation of Innovations in the Healthcare Sector – Practice Through Six Management Perspectives

**DOI:** 10.34172/ijhpm.2021.146

**Published:** 2021-10-25

**Authors:** Klas Palm, Ulrika Persson Fischier

**Affiliations:** Department of Civil and Industrial Engineering, Uppsala University, Uppsala, Sweden.

**Keywords:** Innovation, Implementation, Management, Practice

## Abstract

**Background:** There is a growing expectation that many health organisations will implement innovations. One obstacle for innovative ideas to have an impact on the healthcare system in practice seems to be difficulties in the implementation phase. There is a lack of concretization of theoretical perspectives related to implementation of innovations. The research question answered by this article is: Which enabling factors can facilitate the specific step of moving from idea generation to implementation in a healthcare context?

**Methods:** The research was carried out with a qualitative action research methodology where the researchers took part in the innovation implementation project. The authors of this article were part of a collaborative innovation implementation project involving approximately 54 practitioners. The project was run by five stakeholders: (1) the Division of Assistive Technology in the Dalarna County Council Regional Healthcare Administration, (2) the Habilitation Division, (3) the Division for Home Care and Social Services in the municipality of Leksand, (4) Dalarna University, and (5) Uppsala University. Through a ‘Pearl growing’ technique six implementation management perspectives were, as a framework, identified and presented for the practitioners. The practitioners worked further to concretize these six perspectives. Data was collected through five workshops and collaborations between the researchers and the practitioners. Data was clustered regarding what the managers want to achieve within these six perspectives (ideal situation) and the main means for reaching this situation.

**Results:** The study underlying this article generated 35 concrete enabling factors for successful innovation implementation, distributed over the initially presented six theoretical perspectives.

**Conclusion:** Concretizing management principles into enabling factors shows, on the one hand, that the theoretical principles have practical value, but on the other that they must be adopted to the specific circumstances of each organization, and that too abstract principles can hardly be operationalized.

## Background

**Key Messages**

** Implications for policy makers**

The results of the study offer a solution to managers in the public and private sectors. The implications are:

- Managers not only verbally shall try to change the attitude towards innovations, but also can create concrete leeway for implementation.
- One way to concretize the strategic work of implementing innovations is to work holistically with six management perspectives; (*a*) collaboration with the beneficiaries (ie, patients and other healthcare stakeholders) for the healthcare effort, (*b*) collaborations with other relevant stakeholders in the implementation process, (*c*) organisational culture, (*d*) human resource management, (*e*) organisational structure and (*f*) resource availability.
- Innovation implementation can be facilitated by adjusting and practice of all or some of the 35 enabling factors presented in this article.

** Implications for the public**

The study behind this article generated 35 concrete enabling factors for successful implementation of new innovative products and services in healthcare. Innovation, that above all, focuses on assistive technology, with technical aids to support good everyday life and support and habilitation. Through this type of concretization, the value of previous researchers’ implementation theories increases. Namely, there are many thoughts already in the past about what helps in implementing new solutions. But many theories are not so practically oriented. This article is more concrete and contributes to the possibility for those who work in healthcare to have easier to absorb and use (implement) new innovative products and services. simply, to improve healthcare.

Healthcare as well as other sectors of society need to be developed to respond to new needs and new opportunities. Weintraub and McKee^1^ note that in several countries, there is a growing expectation that health organisations develop, evaluate, and implement innovations. Guarcello^2^ stresses that healthcare innovation improvements have successfully increased patient life expectancy and quality of life, and made access to care, treatments, and diagnostic-path options easier. Guarcello^2^ also argues that innovation in healthcare generates efficiency and reduces costs. Also other scholars eg, Moreira et al^3^ and Boyer^4^ claim that service and process innovations have a great impact on operational performance.

 One big obstacle for innovative ideas to have an impact on the healthcare system seems to be difficulties in the implementation phase, for new ideas to become implemented as standard routines within the organisation, as several authors have noted. Helfrich^5^ et al states that “*often, complex innovations are adopted with great anticipation only to fail during implementation*.” These obstacles to innovate in the healthcare sector has by Currie et al been identified as the ‘translation gap,’ which has two dimensions: “*the translation of basic and clinical research into ideas and products, the so-called T1 gap; and concern for introducing those ideas and products into clinical practice, the so-called T2 gap*.”^6^ The translation gaps are thus on the one hand, to go from knowledge to innovative solutions (ie, products, processes or services), and on the other from having these solutions to actually using them, problems identified for example in the former CLAHRC, now Applied Research Collaborations initiative in England. Previous research has also noted that there is a general lack of empirical research on enabling factors for the specific step of moving from idea generation to implementation.^7-9^ The aim with this paper is therefore to contribute with knowledge of how practitioners and management can overcome the gap between idea generation and implementation of innovations in the healthcare sector. This article thus deals with some dimensions of what management can do to overcome the ‘T2’ gaps, (ie, create circumstances under which it is possible to enable the organisation to implement and use innovative solutions).

 Furthermore, previous research also states that there is a lack of concretization of theoretical perspectives related to how innovations can be implemented. Nilsen^10^ states that existing theory: “*usually are too generic to provide sufficient detail for guiding an implementation process*.” In line with this, Helfrich et al^5^ argue that “*What is missing is a theoretically informed and empirically grounded framework that explains how the interplay of key organizational factors contributes to implementation effectiveness*.”

 An important perspective on implementation of innovations is the managerial perspective. Choi and Chan^11^ highlight the importance of management support for implementation efficiency and collective acceptance of innovations. West and Anderson^12^ have in a study based on managers at 27 hospitals identified that organizational support for innovation was the strongest predictive factor of implementation of organizational changes. The organization’s management, in turn, is of crucial importance for whether an organization is perceived to provide organizational support.

 In order to move forward and develop practical support for the implementation of innovative solutions, six theoretical perspectives (as described in Table 1) have, through a literature review, been identified as appropriate guiding theories to be concretized (ie, provide a framework) for implementation of innovations in a healthcare context. This study was designed to investigate how these six management perspectives can be applied and concretized to promote the step of moving from innovative ideas to implementing innovations in a healthcare context, by collaborating with practitioners in healthcare to explore their perspectives on this. The term practitioners is used in this article to describe someone involved in a skilled job performed in a healthcare context, ie, both managers and employees. The research question answered by this article is: Which enabling factors can facilitate the specific step of moving from idea generation to implementation in a healthcare context?

**Table 1:** Overall Perspectives Important for Management in Healthcare to Create an Enabling Environment for Innovation Implementation

**In Previous Research, Identified Factors That Affect the Ability to Implement Innovations**								**The way in which this study categorizes those perspectives identified by other scholars into six developable management perspectives**
**Grol,** ^23^ ** Approaches to Changing Clinical Practice**	**May and Finch,** ^25^ **Normalisation Process Theory**	**Harvey and Kitson,** ^27^ **i-PARISH**	**Helfrich et al,** ^7^ ** Determinants of Implementation Effectiveness**	**Metz and Bartly's** ^29^ ** Theory of Active Implementation Frameworks**	** Jacobs et al,** ^28^ ** Predictors of Innovation Implementation in Healthcare**	**Palm and Algehed,** ^30^ ** Enablers of Innovative Quality Development in Public Administration**	**Bolman and Deal,** ^31^ **Core Perspectives on Organisation and Management**	
Adopt to needs of target audience	Collective action, interaction with existing practices and relational integration	Identifying and engaging key stakeholders	The fit between the innovation and the values ​​of innovation users	Not applicable	Enrol patients	Not applicable	Not applicable	Collaboration with the beneficiaries for the healthcare effort
Adopt to needs of target audience	Collective action, interaction with existing practices and relational integration	Working across academic, service and other organisational boundaries	Champions in the organisation promotes the innovation	Not applicable	Not applicable	Internal as well as external networking	Not applicable	Collaborations with other relevant stakeholders in the implementation process; contextual adaptation
Social influences	Organizing social norms	Motivate individuals and teams	Innovation fit with users' values		Implementation climate	Communication of achieved tangible results	The symbolic perspective	Organizational culture, symbolic perspectives
Structures for learning, social motivation, dissemination of information	Skill set	Running workshops and advanced master classes on facilitation approaches	Not applicable	Implementation stage, installation: prepare staff and implementation support	Support	Not applicable	The human resource management perspective	Human resource management
Organisational conditions, local consensus development, rational decision making	Organizing structure	Plan, implement, measuring and embedded changes	Formal organisational actions, implementation policies	Implementation stage installation: prepare organisation and use policy and practice loops	Organizational size and structure	Innovation processes alternately organised as a separate project, and as part of the standard operating procedures	The structural perspective	Organisational structure
Economy	Not applicable	Negotiating, competing tensions and manage these	Financial resource availability	Implementation stage installation: resources	Available resources	Not applicable	The political perspective	Resource availability, internal political perspectives
Not applicable	Coherence	Not applicable	The innovation is perceived as an organizational priority by target organizational members	Not applicable	Incentives	Committed hands on leadership and understanding of how parts contributes to a shared vision	Not applicable	Describes the importance of being clear about why the organization needs to implement a new solution, but does not directly provide any input to answer the question of how the innovation should be implemented

###  Innovation

 Lynn^6^ states that: *“Innovation must not simply be another name for change, or for improvement, or even for doing something new lest almost anything qualifies as innovation*.*..*.*”* In line with this, we argue that innovation should not simply be a normative word to denote change in healthcare, but rather to drive increased value in healthcare. In this article, the following definition of innovation is used: innovation is the process of transforming and implementing new ideas into new products, service or processes in order to achieve increased value for patients and citizens.

 The unique value of the concept of innovation lies in the fact that it defines a different process than working with the development of existing products, processes or services. Innovation can be described as exploring new solutions in contrast to incremental development exploiting existing solutions. Exploration is about generating novel recombination’s of knowledge.^13-15^ Exploitation, on the other hand, is created by refinement, efficiency, convergent thinking, and continuous improvement.^15,16^ In this article, we focus on innovation (exploration) of something new; as it may be argued that innovations, not merely incremental improvements (exploitation), are needed to deal with the challenges the healthcare sector is now facing to be able to meet the needs of the future (Guarcello^2^).

 Innovation encounters a challenge that incremental improvements do not face to the same extent; a challenge when moving from idea to implementation. The process can be divided into many or few phases. In this article, we use the simplest form in accordance with Moore & Hartley^17^ and Somech & Drach-Zahavy^18^ who argue that the innovation process in the most basic form can be simplified into two phases – the idea generation phase and the implementation phase. They further argue that innovation occurs only when new ideas and practices are brought into implementation.

###  Management

 In order to create leeway for innovation, the management perspective has been pointed out as crucial by many authors.^2,11,19-21^ Weintraub and McKee^1^ stress that leadership always is important at all levels of the health sector. Also Helfrich et al^5^ argue that management support is a crucial factor in the success of implementing innovations in healthcare. Grol^22^ presents what he calls approaches to ‘changing clinical practice,’ and we agree with this view of the importance of change management as one of the most important factors for creating leeway for innovation.

 This article thus describes implementation theories that management in healthcare can apply as change management tools. One example of an attempt to systematize a theory of change, that healthcare management has used, is May’s^23^ theory called Normalization Process Theory (NPT). It enables analysis of the conditions necessary to support the introduction of complex interventions. May^23^ argue that the NPT theory can be used to understand how a normalization of new techniques and technologies can be done in a healthcare context. Furthermore, May and Finch^24^ state that “*NPT provides a set of sociological tools to understand and explain the social processes that frame the implementation of material practices*.*”* Thus, it is worth noting that they write that NPT is useful for understanding and explaining, but not for implementing new solutions. May and Finch^24^ further state that “*The work of implementation is operationalized through four generative mechanisms (coherence; cognitive participation; collective action; reflexive monitoring).” *May and Finch show that these perspectives emphasize important mechanisms to work with, but do not provide any major guidance for how the innovation leader can work to manage these mechanisms.^25^ May and Finch^24^ themselves believe that “*the theory provides a robust and replicable ecological framework for analyzing the dynamic collective work and relationships involved in the implementation and social shaping of practice*.” Thus, they write that NPT is useful for analyzing, but not providing tools for implementation. Carl May has also developed an NPT toolkit to enable health professionals to use the theory, but the toolkit is still at a relatively high level of abstraction and is suitable for “thinking through” an implementation process or to assess an implementation process (http://www.normalizationprocess.org).

 There are several other attempts at theorizing implementation. One is PARISH, which Harvey and Kitson^26^ describe was developed as early as 1998, but which also has been criticized on the basis “*that implementation misses certain key dimensions, that implementation takes place in a social, political, policy and economic context*.” Harvey and Kitson developed an alternative mode they call i-PARISH, but this theory does not provide clear support in innovation implementation either. Helfrich et al,^5^ presents another framework that addresses the determinants of implementation of complex innovations, including two factors anticipated to be of particular importance in the highly professionalized healthsector: (1) the presence of an innovation champion; and (2) the fit between the innovation and the values ​​of innovation users. Jacobs et al^27^ point out the factors of Climate, Policies, Practices and Enrollment of patients as important for creating conditions for good implementation. Further, Metz and Bartley^28^ claim that there are four important implementation frameworks to deal with: Implementation Stages; Implementation Drivers; Policy– Practice Feedback Loops; and Organized, Expert Implementation Support.

 Worth noting is also that several of these theories, such as NPT and Metz and Bartly’s^28^ theory of Active Implementation Frameworks, are based on challenges in implementing research results and knowledge, which may be different from implementing innovative solutions to perceived challenge, which is in focus of this article.

 In addition to these perspectives from the healthcare context, there exists other implementation research from other empirical fields that point out similar enabling factors for organizational development processes. Palm and Algehed^29^ have determined which – out of a wide range of enabling factors for innovations – may be the most important for the specific process step of moving from ideas to the implementation of innovations in service providing contexts, of which the healthcare sector is one. They argue that five factors can be highlighted as important for the specific innovation process stage of moving from idea generation to implemented innovation. These factors are^29^: (1) a committed and hands-on leadership, (2) a system understanding, including an understanding of how the parts contribute to a shared vision, (3) communication of achieved tangible results, (4) internal as well as external networking and (5) innovation processes alternately organised as a separate project, and as part of the standard operating procedures.

 Furthermore, Bolman and Deal^30^ have distilled theories of organisations to elicit core perspectives on organisation and management. Based on this they presented a theory of four frames in 1984, which contains tools for managers. Bolman and Deals’^30^ perspectives have also been used as reference literature in a number of articles and academic research, both in the management of healthcare^31,32^ and in other studies and business development processes.^33-35^ They identified four important perspectives that provide effective tools for those who need to carry out an organisational development process. These perspectives are (1) the structural perspective, (2) the human resource management perspective, (3) the political perspective, and (4) the symbolic perspective. These perspectives have more of a concrete content in which one can carve out an organizational development process.

 Bolman and Deal’s framework seems also to harmonize with several of these different perspectives described above. Thus, on the basis of the above discussion of various perspectives, both specifically in the health sector, in public administration and general organizational theory, it could be possible to identify certain common perspectives that are important for management in healthcare for creating an enabling environment for innovation implementation. Just like Grol states that implementation seldom entails a single action, but demands a combination of different interventions, it seems that implementation requires simultaneously processing multiple perspectives. For purposes of the underling study discussed in this article, the intervention perspectives identified by previous scholars, were categorized by the researchers into the six perspectives described in Table 1. This table should not be seen as the only possible way to categorize important factors as there is overlap between some of the theories and frameworks and that different authors describe the phenomenon implementation from completely different perspectives.

 For this study, these six perspectives were discussed, tested and validated through a face validity process^36^ with practitioners within the healthcare sector. Their perspectives regarding plausibility and relevance were assessed based on relevant practitioners’ experiences resulting in these perspectives being considered relevant and useful for the execution of our project.

## Methods

**Figure 1:**
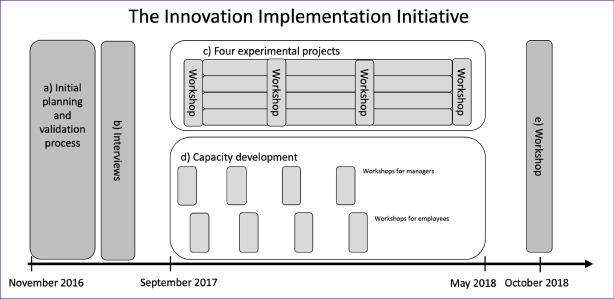
A Description of the Parts in III.

 The project upon which the data collection of this article is based, has been developed from the idea of creating a participatory action research process in which healthcare practitioners could fill theoretical management perspectives with practical content for successful implementation of innovations. This because action research as a method is suitable for not only answering research questions and in that way generate new knowledge, but also at the same time generate a utilization of the new knowledge. Denscombe^37^ argues that implementing action research means that researchers must be practically involved in what is being investigated.In line with Denscombe’s reasoning, we as researchers led and developed all five work packages (a. through e) in III as depicted in Figure 1. The study is carried out through a qualitative method, as that is most suited to gain insight into managers´ views, which is the research question we set out to answer.

 The technique for identifying important perspectives for management in healthcare to create an enabling environment for innovation implementation has been ‘Pearl growing’^38^ (also known as ‘Citation mining’ or ‘Snowballing’). It is an effective approach to systematic literature searching which helps to ensure that relevant literature has been identified.^39^

###  Action Research

 Reason and Bradbury^40^ stress that action research generally supports participants in the research process in solving their own problems. Stringer^41^ describes that action research might violate conventional research methods by not splitting up the relationship between the researcher and the researched objects in a classical way. However, Stringer^41^ further claims that action research supports the participants in the research process to increase their understanding of what is being researched and their own situation. Chevalier and Buckles^42^ argue that action research today should be seen as an important method in professional business development. Through dialogue and development of a common and new narrative new possibilities and conditions for action can be developed.^43^ Aligned with this, also Collins and Hansen^44^ described that adopting dialogic practices to a high extent correlates with probabilities of success in change processes. One dialogue-based development theory is the Dialogic Organizational Development (DOD) theory. Previous research has shown that the DOD has clear benefits for achieving concrete change results.^45,46^ Bushe and Marshak^47^ argue that consultants and researchers in a DOD process should not distance themselves from the development process, but should rather form part of the development process, in order to contribute to real development through dialogue.

###  The Innovation Implementation Initiative as Case

 We used a case study methodology. The case is a healthcare project in the county of Dalarna in Sweden. The research started with the six theoretical perspectives and went on to chisel out the concrete enabling factors for innovation implementation in the healthcare sector in collaboration with managers. The research initiative formed part of an innovation project called the Innovation Implementation Initiative (III) as described in Figure 1. The III was carried out over 24 months, from November 2016 to October 2018. The project was a collaboration between five stakeholders: (1) the Division of Assistive Technology in the Dalarna County Council Regional Healthcare Administration, (2) the Habilitation Division, (3) the Division for Home Care and Social Services in the municipality of Leksand, (4) Dalarna University and (5) Uppsala University. The aim with III was to develop a management model for an enabling environment for implementation of innovations.

 The III involved approximately 24 managers, 30 employees and three researchers, of whom two are authors of this article. These managers consisted of 14 managers within the above-mentioned organization 1, and five managers from organization 2 and five managers from organization 3. That is, three top managers, ie, managers of the entire organisation, six middle managers and 15 first-line managers. The 30 employees consisted of employees that the managers selected to be part of four working groups with the aim of developing methods for implementing innovations. The criteria for the selection of the employees were that the managers considered them to be interested in, and able to contribute to, developing concrete methods for implementing innovations. These were employees who work with testing, adaptation and meeting with beneficiaries (ie, patients and other healthcare stakeholders) as well as those responsible for logistics of assisting technologies, as well as performers of the municipality’s care activities in relation to primarily older municipal residents. In this article the term “employees” is used as a collective term for these practitioners not being managers. The term “practitioners” is used when managers and employees are described jointly.

 The first part (a) was an initial planning and validation process. In the face validity process the researchers presented theories and the six above mentioned management perspectives to the managers who were part of the project, as a suggestion of principles to work with in the project. It was agreed that these management perspectives would be the starting point for further work.

 The second part (b) consisted of interviews with 27 employees and 7 middle and top managers within DAT. The interviews were conducted following a semi-structured interview guide. The interview questions are presented in Supplementary file 1.

 The third part (c) was an action-learning driven design process made up of four different experimental projects. The method of these experimental projects was inspired by the guidebook for service design processes in public administration that NESTA, Ideo and Design for Europe published in 2016.^48^ This guide brings together a collection of practical tools and methods for using design in public services. Design thinking is an often-used strategy or process to gain a deep understanding of the beneficiaries and to generate solutions suited to meet their needs. In previous research, service design theory has been identified as a well-functioning method for driving innovative development in general^49-52^ and also specifically in healthcare.^53-55^ Rahemi et al^54^ writes *“Healthcare providers need to understand that successfully implemented design thinking can enhance patient outcomes, clinical practice, and care quality.” *However, design thinking has to a limited extent been tested and evaluated for the implementation phase of innovation specifically. The projects included an: (1) introduction of circularity for mattresses against bedsores, (2) introduction of apparatus for medicine reminder at home, (3) introduction of interpreting services at a distance; and (4) opportunity to sell and organize sales of new forms of assistive technology products.

 The experimental projects in III were initiated in September 2017 and continued until May 2018. The experimental project groups met and worked in workshops with the process facilitators (the authors of this article) on five occasions. Working with implementation was largely about change management within the organisation and in relation to users.

 The fourth part (d) Four capacity development sessions were held with managers within the organisations involved in the project and four sessions were held for employees. The capacity development sessions included educational and discussion elements based on the six management perspectives. The six management perspectives, as described in Table 1, were communicated as a holistic system perspective in which the six parts complement each other.

 The fifth and last part (e) was a final workshop carried out in order to carve out concrete actions that managers can do when innovation should be implemented. The managers from the three healthcare agencies involved participated in this workshop. This workshop was held after the managers had participated in capacity development for two years and some of their employees had been involved in four experimental projects by which the managers had also been influenced. The aim of the final workshop was to identify what the managers wanted to achieve (ideal situation) and the main means for reaching this within the six identified management perspectives. The methodology used can be described as “back casting” and inspiration came from DOD theories regarding the importance of creating generative images about how the organisation will work in the future.^47^ Raw data from the workshop is presented in Supplementary file 2.

###  Data Collection and Analysis

**Figure 2:**
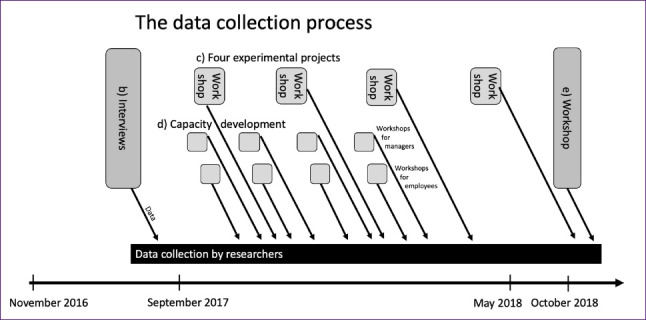
A Description of the Data Collection Process.

 The data collection that forms the basis for this study was generated in part b, c and d and was selected, prioritized and supplemented from the management perspective in the final workshop (part e) in October 2018 (See Figure 2). In the final workshop, ideal states and methods for achieving these states was documented on sticky notes or formulated on white boards in different group rooms and subsequently recorded as a result of the workshop. After the final workshop the researchers organised and clustered statements generated in the workshop regarding what the managers want to achieve (ideal situation) and the main means for reaching this situation within the six identified management perspectives.

## Results

 This article presents the findings of the III-project based upon a framework using the six management perspectives described in Table 1 to improve the value of healthcare by implementing innovative ideas.Results are below exemplified with quotations from Supplementary file 2, ie, statements pointed out at the final workshop.

###  Collaboration With the Beneficiaries for the Healthcare Effort

 A collaboration with beneficiaries needs to take place in two phases. Partly to adapt the innovative solution to the beneficiaries’ needs and conditions and partly after a prototype of the innovative solution has been introduced, feedback systems are needed where the users’ perception of the innovation is fed back to get patients’ feedback to refine the prototype.

 Proposed ways of achieving this involve practical methods of reaching potential users who want to take part in the implementation process, and try out new innovations. This can be done by inviting beneficiaries, and representatives of the patient group, to constitute a “test panel” in the implementation phase. Such co-operation and co-creation can give quick feedback and information on the advantages, disadvantages and challenges of the innovation we want to implement. Participants can share experiences in forms, through interviews, in discussion groups, or by keep a diary of experiences related to the innovation. One way to reach people with an interest in participating in development work which was mentioned on several occasions in the data collection process was the “Dalarna region’s beneficiary library.” The library consists of contact information for people who have self-reported interest in contributing in various ways to development work in healthcare.

*Quotation from participant A: We should be better at use Region Dalarna’s beneficiary library.*

###  Collaborations With Other Relevant Stakeholders in the Implementation Process

 The theory on the importance of networking emphasises the importance of connecting with actors outside the own organisation, to get ambassadors who speak positively about the innovation, and help create a demand for it.

*Quotation from participant B: Involve and engage key people outside the organization. Key people who can speak well for the new idea in different contexts*.

 It was recognised that cooperation is needed with different actors in different phases of the implementation process. Good collaboration is above all needed with close colleagues from support departments such as Digital Support Department, Legal Department and Human Resource Management Department. External collaboration with other organisations needs to be structured. It was emphasised how important it is to have an open mind.

 It was also stressed that the collaborative process with colleagues from support departments and external stake holders start early in the implementation process. In these collaboration processes, it is also important to create a common understanding of needs and opportunities. Creating a common understanding seems like an easy task, especially when we think that it is about collaborations with parts of our own organisation. But considering the various conditions for different parts of the organisation, it is likely very challenging. It was also pointed out that collaborative meetings will consume a lot of time, and that appropriate time needs to be set aside for this. Expectations, objectives and incentives for different actors should be clarified and discussed early in the process. Even if objectives are not common, it is important to be aware of different stakeholders’ different objectives. It may also be important to discuss division of responsibilities. It is important to take notes, and to make clear what decisions were made and what was merely discussed. It is also good to tune in to how each of the participants in a meeting perceived what was being discussed. In processes that run over a longer period, it is important to have continuous feedback to all actors in the collaboration network regarding how far the process has come.

###  Organisational Culture

 As regards the importance of the culture for innovation within the organisation, the practitioners emphasised that one needs to focus on what would be achieved, more than the ability to be innovative. Furthermore, practitioners should feel encouraged to test new solutions and that failed innovation attempts are not a problem. Everyone should feel confident enough to try new solutions and ideas. Trust is therefore something that practitioners identified as crucial.

*Quotation from participant C: Reduce unjustified fear of making mistakes. Reduce “decision anxiety.”*

 A culture in which everyone feels responsible for development and innovation is important, so that everyone sees their own role in development and does not think that it is only the manager’s responsibility to implement the innovation. This means that an organisational culture is needed in which practitioners ask themselves what they need to do to achieve development and innovation goals as opposed to a culture in which we see a need for development, but assume that the situation cannot be improved.

 Ways of achieving this include viewing situations of innovation failure as learning opportunities, to learn why it went wrong. Who did it (who failed) is not important. Another way of phrasing this is to encourage a willingness to learn from errors, which supports innovation (ie, growth versus fixed orientation of the practitioners). When this practitioners growth mindset is combined with a leadership style where the leader believes in “facilitating learning and developing practitioners” a less risk-averse environment is created which further supports organisational innovation.^1^ The desired culture can be achieved by joint discussion on core concepts for the organisation. What does responsibility, trust, following or breaking rules mean in our organisation? It is important to be proud of trying innovations – regardless of whether they fail or succeed.

###  Human Resource Management

 In order to create good conditions for implementation of innovations, it was recognised that different professional backgrounds, knowledge and experiences are needed. This means that a variety of expert knowledge is required, but also that practitioners need basic knowledge about and understanding for innovation and implementation processes. Both those with lengthy experience, and those with fresh eyes and new ideas on how to solve things are required. This means that occupational groups other than those usually being hired by the organisation might be needed. Knowledge about methods for change and implementation, group processes and change of behaviour should also be requested when recruiting new practitioners.

 Continued education of practitioners is also needed, as everyone needs to be aware of the organisation’s vision, goals and assignments. In addition, it is necessary to have insight and understanding of what these visions and goals mean to you as a practitioner in day-to-day work. As innovations are implemented, employees may also become worried that they will no longer be needed, and competence to deal with these kinds of feelings is also needed. To some degree it is a matter of identity.

 It is also important to be able to show early innovation successes even in the case of a major implementation process. This creates enthusiasm and motivation to carry-on the work for implementing the new innovative solution.

 When charting which competences are needed within the organisation, different time perspectives may be applied: in the present, in the near future and in the more distant future. Clear competence development plans related to new innovative working processes can be developed based on goals for the organisation. These can apply to individuals as well as to working groups as a whole. Based on the charted needs for competencies that exist in the group, when recruiting, it is possible to set up a requirements profile, or define what needs the organisation has, and ensure that it is stated in advertisements when recruiting. External recruiters can also be used, to ensure new thinking when recruiting so that not only the professional groups that have traditionally been recruited continue to be prioritised, but also relevant expert competences (ie, information technology) and that those recruited have the desired innovation-positive mindset.

*Quotation from participant D: How do we capture those who are interested in development? How do we design ads? How do we have a dialogue with the employees? Balance between development and “production”? *

 Idea is to recruit those affected by disabilities that form the focus of the organisation in question, since they are best placed to provide an insight about which innovative solutions need to be implemented to benefit them as beneficiaries.

###  Organisational Structure

 The risk here is that middle managers, responsible for budgets and performance, see no real option to let the employees work with innovation. If the cultural dimension is not coupled with changes at a structural level, it risks resulting in the status quo.

 It was discussed how important it was that when developing an innovation-enabling culture, the whole structure of the organisation follows these ambitions. Objectives and indicators need to be developed and formulated in line with the culturally-expressed ambitions for innovation. This includes dedicated time and resources to work with innovations and to test them.

*Quotation from participant E: [innovation need to be] Included in work-plans, control cards, action plans.*

 The structure should allow the formation of small working groups trying to identify and develop small-scale prototypes tested in a realistic environment. These groups should be able to work in a circular process in collaboration with the beneficiaries and practitioners affected by the innovation.

 When formulating steering documents to include innovation, it may be important to make explicit that it is the attempts at achieving innovation and new ways of working that are appreciated, whether or not they succeed. It is the process of trying innovation that should be measured and applauded, not the results. It was also expressed, not least by employees, that it is important that managers give feedback on proposed ideas, so that employees will be aware of where these ideas are heading.

 It was further proposed that the organisation can develop a routine for how implementation of innovative services and products should be managed. This routine can, for example, dictate that implementation processes be agile, small-scale, involve target groups and have frequent reporting to the work team as a whole. An important part of such a routine is also how to work on scaling up and spreading the use of what has been tested on a small scale. In such work, it is important to have good ambassadors for the innovative idea.

###  Resource Availability 

 It should be pointed out that we mainly here consider resources within the healthcare system, rather than as allocation between industry, government and universities. As initially discussed as the^56^ Regular operating budgets should contain leeway for working hours for innovation and the purchase of products as well as service for testing and implementing new solutions. Resources should be set aside for monitoring and benchmarking with similar implementation processes. To achieve this, innovation-related costs should be planned as an obvious part of the regular budget work.

*Quotation from participant F: Natural budget planning for radical innovation*.

 To motivate this, the costs of *not *implementing innovationsneed to be depicted. With systematic follow-ups and evaluations that take into account health economic aspects as a result of the innovation, it can be easier to justify costs associated with the innovation. To carry out follow-ups and evaluations, one can take the help of universities in the form of master’s theses or even doctoral dissertations.

 Although budgeting for innovation in the regular budget work is the most important factor in ensuring a stable financing of innovation processes, it can be a well-functioning method to apply for external financing of implementation processes as well. Grants can also be applied to research, thereby creating even better conditions for future implementation of innovative solutions.

 The findings are summarized in Table 2.

**Table 2:** Summary of Practitioners Perceived Desirable Situations and Methods to Achieve These Desirable Situations

**Six Management Perspective**	**Desired, Ideal Situation **	**Methods to Reach That Desired, Ideal Situation**
Collaboration with the beneficiaries for the health- care effort	Certainty that the innovations address the needs of the beneficiaries already at the point of implementation	Establish and use a “beneficiary library” (ie, beneficiaries who want to be involved in developing healthcare)
		In dialogue discussed new service providing ideas with the beneficiary group
		Inviting beneficiaries, and representatives of the patient group, to constitute a “test panel” in the implementation phase
		With beneficiaries refine designed prototypes. This involves a dialogue over time
		Encouraged beneficiaries to keep a diary of experiences related to the implemented innovation
Collaborations with other relevant stakeholders in the implementation process	Implementation works smoothly within the own organization, thanks to common understanding among the different actors within the own organisation	To early get collaborative partners into the implementation process
		Realize that cooperation is needed with different actors in different phases of the implementation process
		Good collaboration is above all needed with close colleagues from support departments such as IT, Law and Human Resources Department
		Expectations, objectives and incentives for different stakeholders should be clarified and discussed early in the process
		Establish a clear division of responsibilities in the implementation process
		In meetings make clear what decisions were made and what was merely discussed
		Continuous feedback to all actors in the collaboration network regarding how far the process has come
		Develop ambassadors who speak positively about the innovation
		An open mind and curiosity
Organisational culture	Employees try to innovate, sometimes successfully, sometimes not	Focus on what would be achieved, more than the ability to be innovative
		Trust
		Establish a culture where everyone feels responsible for the development of the business
		See eventual innovation failure as learning opportunities, to learn why it went wrong. Create a culture where practitioners feel a willingness to learn from mistakes
		Implement joint discussion on core concepts that arise through the implementation of the new innovation
		Also discuss what concepts such as responsibility, trust, following or breaking rules mean in the organization
		Create a sense of pride of trying innovations - regardless of whether they fail or succeed
Human Resource Management	A multitude of different competences exist in the organisation that can be deployed in innovation work	Recruit new practitioners who have knowledge about methods for change and implementation, group processes and change of behavior
		All practitioners need to be aware of the organization’s vision, goals and assignments
		Strategy and practice for dealing with questioning of identity and professional roles
		Early in the process show positive results, especially in the case of larger and slow implementation processes
		Recruit practitioners from the beneficiaries for the business
Organisational structure	Innovation work can realistically be carried through within the daily work in the organization	Objectives and indicators need to be developed and formulated in line with the culturally-expressed ambitions for innovation
		Develop a routine for how implementation of innovative services and products should be managed
		Form small working groups trying to identify and develop small-scale prototypes tested in a realistic environment
		Implement through a circular iterative process
		Develop a routine how to work on scaling up and spreading the use
Resource availability	Budgets contain leeway for innovation	Resources should be set aside for monitoring and benchmarking with similar implementation processes
		Resources for implementation should be an obvious part of the regular budget
		Plan for evaluations that take into account health economic aspects as a result of the innovation
		The costs of *not *implementing innovations need to be depicted

## Discussion

 In this article, we selected six management perspectives on change management, innovation and implementation as a starting point, and let managers with the practitioners’ experience “fill” them with concrete empirical details on what is needed to enable successful innovation implementation as described by both Nilsen^10^ and Helfrich et al^5^ ask for. The way managers “filled” the management perspectives, can perhaps be seen a way that the four generative mechanisms coherence, cognitive participation, collective action and reflexive monitoring that May and Finch^24^ identify within NPT. However, we can also see that the managers realize the social, political, policy and economic context in which implementation takes place, that Harvey and Kitson^26^ see as usually missing in NPT, similar to the factors of climate, policies and practices that Jacobs et al^27^ describes, and Helfrichs et al^5^ discuss such as Management support, Implementation Policies and Practices and Innovation champions.

 There are several implications for managers to be drawn from our research. Perhaps most importantly, to see how generic management perspectives for innovation and implementation can be concretized. We emphasize the importance of tailoring an implementation strategy based on each organization’s unique contextual conditions. An implementation strategy which may well be based on six important perspectives which are also suitably handled in parallel. By translating the six perspectives, important for implementation, into a concrete and practical change in each unique context, good conditions arise for the implementation of innovative solutions. But without this concretization, these six perspectives do not mean much.

 Based on our study, we suggest that the kind of methodologies we used, DOD and back casting, may be fruitful in implementation processes. These methodologies encourage the development of constructive visions of the future, which is so fundamental for innovation. A more traditional emphasis on “problems” may make stakeholders focus on different aspects, which hinders, rather than enables, innovation. To use DOD and Backcasting can thus be a way to deal with the dilemma of innovation, by focusing on visions rather than problems, but still do it within the ordinary internal work of the organisation, so that both innovations are achieved, but with optimal conditions for implementation as well, as a way to deal with the difficulty of implementation that Helfrich et al^5^ describes.

###  Limitations

 In the research presented in this article, we have used design thinking as a method for generating data. As design thinking to a limited extent has been tested and evaluated for the implementation phase of innovation, we are not sure that the chosen method has been optimal for generating data in response to research questions. The study is done within a part of the healthcare system that is not necessarily representative for all parts of the healthcare system. Different organizations also create different conditions for concretizing general implementation theories. In addition, the closer the analysis is made to concrete factors for implementing innovations, the more context-specific the results can become. This may limit the generalizability. The study has been based on six perspectives that we have sought to concretize through participatory action research. If we had started from different perspectives, the result could possibly have been different.

## Conclusion

 The research question in this article is: Which enabling factors can facilitate the specific step of moving from idea generation to innovation implementation in a healthcare context? The answer is that practitioners need to tailor an implementation strategy based on generic principles and theories about implementation and adjust and practice all or some of the 35 concrete means described in Table 2 divided into six categories of change (management perspectives) presented in this article; (1) collaboration with the beneficiaries for the health- care effort, (2) collaborations with other relevant stakeholders in the implementation process, (3) organisational culture, (4) human resource management, (5) organisational structure and (6) resource availability.

 One of the single most important conclusions is that it is also important that managers do not talk about innovation, but about what generative images one has of the future. It is also extremely important that one not only tries to culturally change the attitude towards innovation, but also create the structural conditions for innovation. Simply talking about how important it is, but not creating structural space can lead to management being accused of paying lip service. However, continued research should be conducted on how implementation theories can be concretized in different healthcare contexts.

## Ethical issues

 The study does not involve any physical or mental intervention on any individual or uses a method that aims to influence the respondents physically or mentally. The study does not include any processing of sensitive personal data. This research does not contain any personal information. The project behind this article has been conducted in dialogue with practitioners at various levels and in dialogue with concerned practitioners. Representatives of participating organizations have been involved in both writing the application for funding the III project and designing of the research process. The participants in the project have voluntarily and in open seminars shared their opinions and experiences. The participants have had opportunity not to communicate such data they did not want to share. The study has been conducted through a participatory action research method, ie, the research has been carried out together with the departments and practitioners concerned. The participants have known that they contribute to the identification of success factors for business development and implementation of innovative solutions and that they have been part of an action research project. The results of the study have been communicated to and discussed by the participants in the study. Participation has thus taken place with informed consent. These factors described above have led to the assessment that no ethical review was necessary.

## Competing interests

 Authors declare that they have no competing interests.

## Authors’ contributions

 KP and UPF have jointly worked with the design of the study, theory studies, data collection, analysis and writing of the article.

## Funding

 Vinnova, Sweden’s innovation agency.

## Supplementary files

Supplementary file 1. Questionnaire.

Supplementary file 2. Empirical Data Generated in the Concluding Workshop.

## References

[1] Weintraub P, McKee M (2019). Leadership for innovation in healthcare: an exploration. Int J Health Policy Manag.

[2] Guarcello C, de Vargas ER (2020). Service innovation in healthcare: a systematic literature review. Lat Am Bus Rev.

[3] Moreira MRA, Gherman M, Sousa PSA (2017). Does innovation influence the performance of healthcare organizations?. Innovation.

[4] Boyer R (2019). How scientific breakthroughs and social innovations shape the evolution of the healthcare sector.

[5] Helfrich CD, Weiner BJ, McKinney MM, Minasian L (2007). Determinants of implementation effectiveness: adapting a framework for complex innovations. Med Care Res Rev.

[6] Currie G, Lockett A, El Enany N (2013). From what we know to what we do: lessons learned from the translational CLAHRC initiative in England. J Health Serv Res Policy.

[7] Brorström S (2015). Implementing innovative ideas in a city: good solutions on paper but not in practice?. Int J Public Sect Manag.

[8] Stewart J (2014). Implementing an innovative public sector program: the balance between flexibility and control. Int J Public Sect Manag.

[9] Piening EP (2011). Insights into the process dynamics of innovation implementation. Public Manag Rev.

[10] Nilsen P (2015). Making sense of implementation theories, models and frameworks. Implement Sci.

[11] Choi JN, Chang JY (2009). Innovation implementation in the public sector: an integration of institutional and collective dynamics. J Appl Psychol.

[12] West MA, Anderson NR (1996). Innovation in top management teams. J Appl Psychol.

[13] Taylor A, Greve HR (2006). Superman or the fantastic four? knowledge combination and experience in innovative teams. Acad Manage J.

[14] Wadhwa A, Kotha S (2006). Knowledge creation through external venturing: evidence from the telecommunications equipment manufacturing industry. Acad Manage J.

[15] Simsek Z (2009). Organizational ambidexterity: towards a multilevel understanding. J Manag Stud.

[16] March JG (1991). Exploration and exploitation in organizational learning. Organ Sci.

[17] Moore M, Hartley J (2008). Innovations in governance. Public Manag Rev.

[18] Somech A, Drach-Zahavy A (2013). Translating team creativity to innovation implementation: the role of team composition and climate for innovation. J Manage.

[19] Birken SA, Lee SY, Weiner BJ, Chin MH, Chiu M, Schaefer CT (2015). From strategy to action: how top managers’ support increases middle managers’ commitment to innovation implementation in health care organizations. Health Care Manage Rev.

[20] Denti L. Leadership and Innovation in R&D Teams. Gothenburg, Sweden: University of Gothenburg; 2013.

[21] Albury D (2011). Creating the conditions for radical public service innovation. Aust J Public Adm.

[22] Grol R (1997). Personal paper Beliefs and evidence in changing clinical practice. BMJ.

[23] May C (2006). A rational model for assessing and evaluating complex interventions in health care. BMC Health Serv Res.

[24] May C, Finch T (2009). Implementing, embedding, and integrating practices: an outline of normalization process theory. Sociology.

[25] May CR, Finch T, Ballini L (2011). Evaluating complex interventions and health technologies using normalization process theory: development of a simplified approach and web-enabled toolkit. BMC Health Serv Res.

[26] Harvey G, Kitson A (2016). PARIHS revisited: from heuristic to integrated framework for the successful implementation of knowledge into practice. Implement Sci.

[27] Jacobs SR, Weiner BJ, Reeve BB, Hofmann DA, Christian M, Weinberger M (2015). Determining the predictors of innovation implementation in healthcare: a quantitative analysis of implementation effectiveness. BMC Health Serv Res.

[28] Metz A, Bartley L (2012). Active implementation frameworks for program success. Zero Three.

[29] Palm K, Algehed J (2017). Exploring enablers of innovative quality development in public administration. Int J Qual Serv Sci.

[30] Bolman LG, Deal TE. Modern Approaches to Understanding and Managing Organizations. San Francisco: Jossey-Bass; 1984.

[31] Hemman EA (2002). Creating healthcare cultures of patient safety. J Nurs Adm.

[32] Nemec J, Spacek D, de Vries MS (2015). Coordinating healthcare under a pluralistic health insurance system: the case of Slovakia. Transylv Rev Adm Sci.

[33] McKimm J, Jones PK (2018). Twelve tips for applying change models to curriculum design, development and delivery. Med Teach.

[34] Zhitlukhina OG, Babak LN, Rakutko SY (2018). Specificity of the relationship between project management and organizational culture. J Entrep Educ.

[35] Bolman LG, Deal TE. Reframing Organizations: Artistry, Choice, and Leadership. John Wiley & Sons; 2017.

[36] Arbnor I, Bjerke B. Methodology for Creating Business Knowledge. 3rd ed. SAGE Publications; 2009. 10.4135/9780857024473.

[37] Denscombe M. The Good Research Guide: For Small-Scale Social Research Projects. McGraw-Hill Education (UK); 2014.

[38] Hawkins DT, Wagers R (1982). Online bibliographic search strategy development. Online.

[39] Badampudi D, Wohlin C, Petersen K. Experiences from using snowballing and database searches in systematic literature studies. In: Proceedings of the 19th International Conference on Evaluation and Assessment in Software Engineering. Association for Computing Machinery (ACM); 2015. 10.1145/2745802.2745818.

[40] Reason P, Bradbury H. Handbook of Action Research: Participative Inquiry and Practice. SAGE Publications; 2001.

[41] Stringer ET. Action Research: SAGE Publications; 2013.

[42] Chevalier JM, Buckles DJ. Participatory Action Research: Theory and Methods for Engaged Inquiry. Routledge; 2019.

[43] Ford JD, Ford LW (1995). The role of conversations in producing intentional change in organizations. Acad Manage Rev.

[44] Collins J, Hansen MT. Great by Choice: Uncertainty, Chaos and Luck-Why Some Thrive Despite Them All. Random House; 2011.

[45] Vacik H, Kurttila M, Hujala T (2014). Evaluating collaborative planning methods supporting programme-based planning in natural resource management. J Environ Manage.

[46] Bushe GR, Marshak RJ. Dialogic organization development. In: Jones BB, Brazzel M, eds. The NTL Handbook of Organization Development and Change. John Wiley & Sons; 2014:193-211.

[47] Bushe GR, Marshak RJ. The dialogic mindset in organization development. In: Research in Organizational Change and Development. Emerald Group Publishing Limited; 2014.

[48] Nesta and IDEO. Designing Public Services: a practical guide. http://www.nesta.org.uk/publications/designing-public-services-practical-guide. Published 2016.

[49] Cross N (2001). Designerly ways of knowing: design discipline versus design science. Des Issues.

[50] Norman D (2002). Emotion & design: attractive things work better. Interactions.

[51] Bason C. Leading Public Sector Innovation: Co-creating for a Better Society. Policy Press; 2010.

[52] Gaziulusoy Aİ, Ryan C (2017). Shifting conversations for sustainability transitions using participatory design visioning. Des J.

[53] Roberts JP, Fisher TR, Trowbridge MJ, Bent C (2016). A design thinking framework for healthcare management and innovation. Healthcare.

[54] Rahemi Z, DʼAvolio D, Dunphy LM, Rivera A (2018). Shifting management in healthcare: an integrative review of design thinking. Nurs Manage.

[55] Vink J, Edvardsson B, Wetter-Edman K, Tronvoll B (2019). Reshaping mental models–enabling innovation through service design. J Serv Manag.

[56] Etzkowitz H, Leydesdorff L (1995). The triple helix--university-industry-government relations: a laboratory for knowledge based economic development. EASST Rev.

