# Supplementary material for: What Managers Find Important for Implementation of Innovations in the Healthcare Sector – Practice Through Six Management Perspectives
Source: Int J Health Policy Manag. 2021 Oct 25;11(10):2261–71. doi: 10.34172/ijhpm.2021.146 (PMC9808278; doi:10.34172/ijhpm.2021.146)
Supplement: Supplementary file 1 — Questionnaire. [file ijhpm-11-2261-s001.pdf]

Article title: What Managers Find Important for Implementation of Innovations in the Healthcare Sector – Practice Through Six Management Perspectives

Journal name: International Journal of Health Policy and Management (IJHPM)

Authors' information: Klas Palm*, Ulrika Persson Fischier

Department of Civil and Industrial Engineering, Uppsala University, Uppsala, Sweden.

(*Corresponding author: klas.palm@angstrom.uu.se)

Supplementary file 1. Questionnaire

Refers to Figure 1, section: b) Interviews

Interview questions

1. What needs for implementation of new innovations do you see?
2. In order to facilitate the implementation of those innovations, can something be done related to the organization's culture do you think?
3. In order to facilitate the implementation of those innovations, can something be done related to the organization's structure?
4. In order to facilitate the implementation of those innovations, can something be done related to the organization's human resource management?
5. In order to facilitate the implementation of those innovations, can something be done related to how the organization's allocate resources in the organization?
6. In order to facilitate the implementation of those innovations, can something be done related to how the organization strategically and practically collaborates with patients and beneficiaries?
7. In order to facilitate the implementation of those innovations, can something be done related to how the organization strategically and practically collaborates with other main stakeholders?
