# Supplementary material for: What Managers Find Important for Implementation of Innovations in the Healthcare Sector – Practice Through Six Management Perspectives
Source: Int J Health Policy Manag. 2021 Oct 25;11(10):2261–71. doi: 10.34172/ijhpm.2021.146 (PMC9808278; doi:10.34172/ijhpm.2021.146)
Supplement: Supplementary file 2 — Empirical Data Generated in the Concluding Workshop. [file ijhpm-11-2261-s002.pdf]

Article title: What Managers Find Important for Implementation of Innovations in the Healthcare Sector – Practice Through Six Management Perspectives

Journal name: International Journal of Health Policy and Management (IJHPM)

Authors' information: Klas Palm*, Ulrika Persson Fischier

Department of Civil and Industrial Engineering, Uppsala University, Uppsala, Sweden.

(*Corresponding author: klas.palm@angstrom.uu.se)

Supplementary file 2. Empirical Data Generated in the Concluding Workshop

Refers to Figure 1, section: e) Workshop

Compilation of sticky notes and notes on whiteboards made by the working groups during the workshop.

Organisational structure

Working group I

We need:

- Ideas - systems to handle it in. Taken care of by some grouping. Clarity what the process looks like, clarity what feedback looks like.
- Idea generation leeway at workplace meetings, a structure how to handle idea generation.
- An Innovation Council. A council (association of employees-managers) where you can come up with your ideas; help, I hear the thought, I have this frame. 1 line manager's first mandate that it's OK to spend money on different projects (no need to ask about). Test small scale.
- Catalyst. Representative from various units ex municipality and habilitation.
- The idea should not be taken over. The creator of the idea must always be the speaker.
- Must have perspective for 3 years ahead.
- Examples of ideas that are rewarded. (Show examples of implemented innovations that have positively succeeded in contributing to business development).

Working group II

We need:

- Structure is needed to be able to work with when everyday life rolls on.
- Must be given time to be able to talk about development and innovation.
- Take advantage of the time we already have at, for example, various meetings, appoint working groups, test.

- Often an entire chain that becomes dependent / affected by a change. Must think all the way and involve several activities.

The ideal situation:

- An idea comes up on an workplace meeting, under the item idea forum.
- The idea is discussed and you come up with a little different angle.
- The idea matures and is taken up again at the next meeting. You decide to go ahead with the idea.
- A working group is appointed, does anyone outside their own unit need to be involved.
- The manager gives a mandate for the group to set aside some time.
- A schedule is drawn up.
- Reporting on an ongoing basis, are more resources needed? more people involved?
- Final report.
- Then a decision on implementation in the business.

Our various measures

- Encourage employees to come up with ideas and suggestions.
- Set aside time.
- Included in work-plans, control cards, action plans.
- Meetings across "borders".
- Create a freer agenda at meetings.
- Invite a player to regular meetings.
- Today, a "crisis situation" is needed to think new.

<u>Working group III</u>

We need:

- Meeting forum between managers of companies that work with similar patient groups. Open discussions, dare to throw out crazy ideas without thinking in terms of finances, staff, etc.
- Focus on solutions - the question "how do we solve what we face?"
- “Peel the onion” - Why is this a problem? What are you trying to achieve? How would you like to solve it if there were no obstacles?
- Template from the manager's side to give the employee an assignment in innovation - Structure, financial framework, time frame, mandate.
- Involve the right professional categories depending on issues to create discussions and possible innovations at the right level in the organization.

Our various measures

- Encourage employees.
- Set aside time.
- Included in strategy, control card, action plan.
- Meetings across "borders".
- Create a "freer agenda" at meetings.
- Invite a player to regular meetings.
- Today, a crisis situation is needed to think new.

Organisational culture

<u>Working group I</u>

We need:

- Reduce unjustified fear of making mistakes. Reduce “decision anxiety”.
- The 1st line chiefs should have an open mind and receptivety to ideas.
- The 1st line chiefs should have confidence in staff.
- Deal with culture in the right way! The boss's responsibility!
- Clear visions and goals. Follow-up of goals.

Working group II

We need:

Ideal mode

- Encouragement to try, without having a clue how it will be.
- That everyone thinks that it is everyone's responsibility to participate in change work (not just the management's).
- That old negative values / patterns are not transferred to new employees.
- That you ask yourself what you need to reach the goal instead of saying that it is not possible…
- That even an organizational culture can benefit from having a financial framework / budget / routines for different processes.
- "Dare to try".

How?

- The management needs to present a better concept… I see this, how could we do to bring about a change? Invite to participation at an early stage!
- When commissioning to make “cultural change”, the manager should be present… recognize, support, manage and possibly correct.

Human Resource Management

Working group I

- Focus on values.
- Clear competence development plan that takes into account the company's and employees' future needs to meet the target group.
- Skills supply / When recruiting What is really needed?
- The right person in the right place.
- Encourage new thinking.
- Allow innovation in everyday life.
- Take advantage of the creativity of employees.
- Dissemination of good examples.

Working group II

- Linus' three success factors:

    1. It's not about the gear - it's about us (understanding and acceptance).
    2. Coolness.
    3. Who am I for? Why do we do this? Or "curiosity".

- How do we capture those who are interested in development? How do we design ads? How do we have a dialogue with the employees? Balance between development and "production"?
- Dialogue with staff about clear expectations. Direction essential to provide conditions.
- An anchored common set of values and an overall acceptance of "why"?
- Key focus: Security - competence requirements – strengths.

Ideal mode

- Cognitive debriefing around essential concepts - do we see / hear the same thing? The meaning of a concept such as trust or responsibility
- Anchor the overall purpose and vision
- Knowledge of psychology: Group dynamics - change process. First managers but also employees. Understanding and acceptance in all situations that we act and react differently and how we meet each other in it.
- Values - rooted and known to everyone in the business - ex high priority at introduction
- Work for a we feeling - common prio - activities that build cohesion.
- Mapping of competence - needs and what is available: now, 1-2 years 2-7 years and 10 years
- Pay attention to the employees' work (continuous feedback) - reporting of processes and results regarding the ideas that the organisation work with (assumes good system support)
- Discuss and accept the time perspective - we need to accept at all levels that changes take a long time
- Find a structure to work / highlight innovation - point at work-place meetings.
- Diversity and mix….
- Business development processes create the conditions for and are made possible by including them in, for example, routines, organizational solutions. Time must be given to these processes.

As an employee, I have:

- Knowledge of why my job exists.
- A value base anchored in the business.
- A mutually accepted / anchored assignment - which is based on the target group's needs.
- Conditions to carry out the assignment.
- Close dialogue with my "closest" (colleagues, managers, customers / target group).

As an employee, I feel:

- Motivation.
- Security.
- Joy.
- Flow.
- I feel that I am important / needed / an important part of the whole. Like “Sense of Coherence", SOC.

<u>Working group III</u>

- Cognitive debriefing around essential concepts.
- Anchor the overall purpose and vision.
- Knowledge of psychology: group dynamics, change.
- Values that are known to employees.
- Anchor values.
- "We-feeling" - common prioritization.

- Map competence NOW; for 1 - 2 years, 2-7 years and 10 years.
- Pay attention to the employee's work.
- Reporting of processes and results regarding ideas.
- Anchor time perspectives - acceptance that things take time.
- It has to be a part of everyday life, (e.g., a fixed point on the workplace meeting agenda).
- Working groups on development ideas - common priority.
- Continuing education, training.
- Manager / leader - lead by example "live as we learn".
- Flexibility.

Resource availability

<u>Working group I</u>

Keywords:

- Natural budget planning for radical innovation.
- Part of the systematic budget work. Provides clarity and space.
- Allocated funds at County Council and municipal level.
- At the administrative level, the county council / sector level municipality should have an innovation council.
- The council must make decisions and distribute an “innovation pot” / allocated funds for radical innovation.
- The ideas can come from all employees, but with centrally allocated funds, conditions are created for 1st line managers to allocate staff for the systematic innovation work.
- It must be possible to take in substitutes.
- Economic space is a prerequisite for other system perspectives.

<u>Working group II</u>

- Instruments for easy monitoring of finances.
- Follow-up / evaluation.
- Clear picture where possible finances for innovation are.
- "Innovation of the year".
- Long-term financial plan.
- Experts from other businesses.
- Exchange of experience within and outside the own business.
- Sees the socio-economic effects.

Collaborations with other relevant stakeholders in the implementation process

<u>Working group II</u>

Ideal Mode

- Flexible forms of meetings.
- Feedback to indirect groups.

- Key people.
- Clarification: discussion or decision.
- Memoirs.
- Rules for collaboration.
- Time frames.
- Clarify expectations.
- Clarify Purpose and goal.
- Probe the terrain.
- Involve expertise.
- Trust and respect.
- It is extremely important to involve the “support departments”.
- Low prestige.

Means

- Trust and respect, leads naturally to collaboration.
- In the event of a change, it is important to involve different groups / knowledge to make it easier to capture any obstacles.
- Involve stakeholders early, (they who are affected).
- Collaboration and accessibility.
- Purpose and goals are important in collaboration - are the key people involved?
- Clarity, was it a decision or how did everyone perceive it? Summary at the end of the meeting… we have perceived it in the same way.
- Preparation / probing the terrain - workplace meeting can be a good start to check the situation, which groups we are in, what we want to work with, which are interested.
- Someone clarifies the purpose and goals of the grouping and summarizes at the end
- Discuss expectations and rules of conduct. How do we respond to emails?
- If the process is longer, it is important to have continuous feedback to others about how far the process has come. (In workplace meetings and other forums).
- Low prestige - that we can bring in expert knowledge when needed.
- Flexible meeting forms - Intra, Skype, video conference, meeting.
- Clarify limits of liability.

<u>Working group III</u>

Ideal image

- Openness and curiosity for each party's situation.
- Respect and acceptance because we are different and have different assignments.
- Established communication channels between different organizations and principals.
- Know who is involved at an early stage. How do we synchronize? "start at the same time".
- Understanding why we collaborate - purpose of collaboration.
- The leadership should not matter.
- We understand that change takes time and that we stand in this together.
- We have the conditions locally to collaborate in a larger perspective.

How do we reach the ideal image?

- Consultation through an assignment (not ad hoc) - e.g., creating knowledge.

- Set aside time to participate in a form of collaboration.
- Create forums to develop relationships between partners - bring together different competencies (the Medici Effect).
- Involve and engage key people outside the organization. Key people who can speak well for the new idea in different contexts.
- Encourage to try - start on a smaller scale (dare to test).
- Lead by example as a manager - show a working form of collaboration.

Collaboration with the beneficiaries for the health care effort

Working group I

Our employee meets a user / patient / citizen when prescribing aids. The ideal situation is to obtain the user's story at the meeting. Obtain information about resources and conditions / support the person has in everyday life. Based on that, we offer what we have for services and technology. Based on that, agree / enter into a partnership and agree on what the person should do / perform and what we should do / perform. It is important that it is documented in a plan. Follow-up is important to see if the aid is used or if it is actually another need of the user / patient. Collaborate / collaborate with others internally / externally if needed, there are no financial aspects between the units.

Working group II

Keywords in the target group perspective:

- Cognitive impairment - living at home longer with aids.
- Teamwork.
- That citizens are involved with their ideas.

Working group III

- Participation.
- Cognitive impairment - living at home longer with aids.
- Ethics.
- Collaboration with the network around the customer / patient.
- Systematic dialogue.
- “Pre” target group involved in the early stages.
- Participating citizens – ideas.
- New construction.
- Listen and use their life stories.
- It is about economics…
- New meeting cultures, forums.
- Involve beneficiaries in two phases; when adjusting the innovation and when the innovation is really used.
- Environmental monitoring.
- Permissible organizational structure.
- More user forums to test prototypes.
- More person-centered approach.
- We should be better at use Region Dalarna’s beneficiary library.
